# Endoscopic Treatment of Superficial Gastric Cancer: Present Status and Future

**DOI:** 10.3390/curroncol29070371

**Published:** 2022-07-04

**Authors:** Hiroyuki Hisada, Yoshiki Sakaguchi, Kaori Oshio, Satoru Mizutani, Hideki Nakagawa, Junichi Sato, Dai Kubota, Miho Obata, Rina Cho, Sayaka Nagao, Yuko Miura, Hiroya Mizutani, Daisuke Ohki, Seiichi Yakabi, Yu Takahashi, Naomi Kakushima, Yosuke Tsuji, Nobutake Yamamichi, Mitsuhiro Fujishiro

**Affiliations:** Department of Gastroenterology, The University of Tokyo Hospital, 7-3-1, Hongo, Bunkyo-ku, Tokyo 113-8655, Japan; hisadah-int@h.u-tokyo.ac.jp (H.H.); oshiok-int@h.u-tokyo.ac.jp (K.O.); mizutanis-int@h.u-tokyo.ac.jp (S.M.); nakagawahi-int@h.u-tokyo.ac.jp (H.N.); satoju-int@h.u-tokyo.ac.jp (J.S.); kubotad-int@h.u-tokyo.ac.jp (D.K.); obatam-int@h.u-tokyo.ac.jp (M.O.); chor-int@h.u-tokyo.ac.jp (R.C.); nagaos-int@h.u-tokyo.ac.jp (S.N.); miuray-int@h.u-tokyo.ac.jp (Y.M.); mizutanih-int@h.u-tokyo.ac.jp (H.M.); okid-int@h.u-tokyo.ac.jp (D.O.); yakabis-int@h.u-tokyo.ac.jp (S.Y.); takahashiyu-int@h.u-tokyo.ac.jp (Y.T.); kakushiman-int@h.u-tokyo.ac.jp (N.K.); tsujiy-int@h.u-tokyo.ac.jp (Y.T.); yamamichin-int@h.u-tokyo.ac.jp (N.Y.); fujishiromi-int@h.u-tokyo.ac.jp (M.F.)

**Keywords:** gastric cancer, endoscopic diagnosis, endoscopic submucosal dissection, endoscopic resection, curability assessment, endoscopic screening

## Abstract

Although the mortality rates of gastric cancer (GC) are gradually declining, gastric cancer is still the fourth leading cause of cancer-related death worldwide. This may be due to the high rate of patients who are diagnosed with GC at advanced stages. However, in countries such as Japan with endoscopic screening systems, more than half of GCs are discovered at an early stage, enabling endoscopic resection (ER). Especially after the introduction of endoscopic submucosal dissection (ESD) in Japan around 2000, a high en bloc resection rate allowing pathological assessment of margin and depth has become possible. While ER is a diagnostic method of treatment and may not always be curative, it is widely accepted as standard treatment because it is less invasive than surgery and can provide an accurate diagnosis for deciding whether additional surgery is necessary. The curability of ER is currently assessed by the completeness of primary tumor removal and the possibility of lymph node metastasis. This review introduces methods, indications, and curability criteria for ER of EGC. Despite recent advances, several problems remain unsolved. This review will also outline the latest evidence concerning future issues.

## 1. Introduction

Gastric cancer (GC) is the fifth most common cancer and the fourth leading cause of cancer-related death worldwide [1]. *Helicobacter pylori (H. pylori)* infection is the leading risk factor for GC, and thus GC occurs mainly in Asia and Eastern Europe, where there is a high prevalence of *H. pylori* infection [1]. Other factors, including tobacco smoking, alcohol, and salted food consumption, are also known to be risk factors for GC but, compared to *H. pylori* infection, have a much more limited influence on GC prevalence [2,3,4]. In Japan and South Korea, which are two of the leading countries in incidence of GC, following China, GC screening programs on a national level have been implemented with radiographic (X-ray) examination or gastroscopy recommended at annual or biannual intervals [5]. These screening programs have been reported to increase the detection rate of early gastric cancer (EGC) and prevent GC-related mortality [6,7]. This, in turn, enables minimally invasive treatment with endoscopic resection (ER), which is now accepted as first-line management for most EGC. ER includes widely performed methods such as endoscopic mucosal resection (EMR) and endoscopic submucosal dissection (ESD), with ESD being the method of treatment most recommended in current guidelines for EGC [8,9]. This review outlines the status and future prospects of endoscopic diagnosis and treatment for GC.

## 2. Endoscopic Screening and Diagnosis

### 2.1. Endoscopic Screening for Discovering Early Gastric Cancer

Despite the high prevalence and high cancer-related death rate of GC worldwide, there is no international standard for screening for GC, with the exception of Japan and South Korea. For example, national guidelines in Japan for GC screening recommend annual X-ray screening from the age of 40 years or biennial endoscopic screening from 50 years [5]. Though only an X-ray examination was conducted for screening for GC until 2016, endoscopic screening was added to GC screening due to the evidence of a lower rate of gastric cancer-related death and a higher detection rate of EGC in endoscopic screening in Japan than X-ray examination [10,11,12]. Similarly, Korean national guidelines for GC screening recommend endoscopic screening for subjects aged 40 years or over [13]. Recent reports on these screening programs have focused on the need for quality indicators during endoscopic screening, with previous reports suggesting that sufficient observation time is important for improving the detection of GC [14]. Although there may still be room for improvement in the quality and permeation of GC screening, these screening programs have already been shown to greatly reduce the death rate from GC [10,11,15].

### 2.2. Indications of Endoscopic Resection for Gastric Cancer

Whether a lesion is indicated for ER is determined by the risk of lymph node metastasis and whether it can be resected en bloc [8]. Initially, the Japanese gastric cancer treatment guidelines, first edition, which was the first guideline to determine the indication for ER of GC, defined the indication for ER as well-differentiated intramucosal cancers of less than 20 mm diameter without ulceration findings (UL0).

Thereafter, studies examining the long-term prognosis of ESD have accumulated, and currently, indications for ER are divided into absolute indications, expanded indications, and relative indications, which are defined as follows [16,17,18,19,20].

Absolute indications are defined as lesions with an estimated risk of lymph node metastasis of less than 1% and with equal long-term outcomes to surgical resection (Table 1) [8,21]. Expanded indications are defined as lesions with an estimated risk of lymph node metastasis of less than 1% but with poor evidence equal to surgical resection in terms of long-term outcomes. Relative indications are defined as lesions other than absolute indications or extended indications and may be indicated based on the possibility of curative endoscopic treatment due to the uncertainty of preoperative endoscopic diagnosis and the patient’s general condition.

### 2.3. Assessment of Horizontal Extent and Depth Diagnosis of Gastric Cancer

Following the detection of GC, the selection of optimal treatment methods is required. Especially in cases of EGC, accurate endoscopic diagnosis of the horizontal extent and depth is essential for deciding indications for ER. While the primary diagnosis of EGC is performed with white light observation, which is available at most health care centers with endoscopy equipment, the efficacy of more detailed endoscopic examination at tertiary referral centers to determine indications for treatment has also been reported [8]. Magnifying endoscopy with narrow-band imaging and chromoendoscopy are two methods that have been reported to improve the accuracy of diagnosis of horizontal extent in EGC, with magnifying endoscopy having higher accuracy rates [22]. The most widely accepted algorithms for the differential diagnosis and assessment of the horizontal extent of EGC are based on the Magnifying Endoscopy Simple Diagnostic Algorithm for Gastric cancer (MESDA-G) [23]. In the MESDA-G algorithm, evaluation of vessels and surface patterns with magnifying endoscopy with NBI is performed. The existence of a demarcation line (DL), which is an abrupt change in these patterns, leads to a diagnosis of EGC, with accuracy rates of up to 97% [24]. However, a clear DL cannot always be distinguished in undifferentiated type cancer because the type infiltrates beneath the surface epithelium [25]. Therefore, biopsy samples from surrounding areas adjacent to the endoscopically detectable cancerous region are recommended in lesions of undifferentiated intramucosal cancers in order to accurately assess the horizontal extent of these lesions [22,25].

Depth diagnosis of EGC, especially differentiation of submucosal invasion of more than 500 µm (T1b2), is also essential in the decision-making process of whether to perform ER for EGC. Macroscopic endoscopic findings such as tumor size > 30 mm, remarkable redness, uneven surface, submucosal tumorlike margin elevation, and mucosal fold convergence are reported to be useful in the depth diagnosis of EGC. The accuracy of depth diagnosis using those findings is reported to be 82.5–96.9% [26,27,28]. While these findings are subjective and may be influenced by the endoscopists’ experience, similar accuracy rates have been reported in cases diagnosed by experts in various high-volume centers, demonstrating the clinical efficacy of this method.

Another method for the depth diagnosis of GC is endoscopic ultrasonography (EUS); however, at present, the efficacy of EUS is controversial [29]. In a retrospective study to compare the accuracy of EUS and macroscopic findings in conventional endoscopy for depth diagnosis of GC, conventional endoscopy was more useful for depth diagnosis than EUS; 73.7% vs. 67.4%, *p* < 0.001 [30]. Moreover, ulcer findings have been shown to reduce the diagnostic accuracy of EUS [31]. In addition, although EUS enables an objective assessment of the invasion depth of GC, this advantage is counterbalanced by the disadvantages of additional time and costs of performing EUS. Thus, because of the limited accuracy and additional burden of performing EUS, it is recommended that depth diagnosis be made by conventional endoscopy and EUS be used as a secondary method only for selective cases [8,21].

### 2.4. History of Endoscopic Resection for Gastric Cancer

The history of endoscopic resection for GC dates back to 1974 when Oguro et al. reported the first polypectomy treatment for EGC [32]. The introduction of polypectomy enabled ER of gastric lesions, which previously could only be resected through radical surgery, and was a turning point in the management of EGC. However, while polypectomy enabled ER of gastric polypoid lesions, technical difficulties for ER of flat lesions remained. This problem was greatly resolved when Tada et al. reported the first EMR treatment for EGC in 1983 [33]. However, even with the introduction of EMR, the size of lesions that could be resected en bloc was limited. The first ESD to treat EGC was first described in the 1990s [34,35,36], enabling en bloc resection of lesions larger than 2 cm. The en bloc resection rate, histologic complete resection rate, and curative resection rate in ESD have been reported to be significantly higher than EMR, with the local recurrence rate in ESD significantly lower than EMR [37,38]. As a result, the European Society of Gastrointestinal Endoscopy (ESGE) and Japanese guidelines recommend ESD as the first-line treatment of EGC [8,9,21]. 

### 2.5. Methods of Endoscopic Resection for Gastric Cancer

#### ESD (How to Perform) 

**Figure 1:**
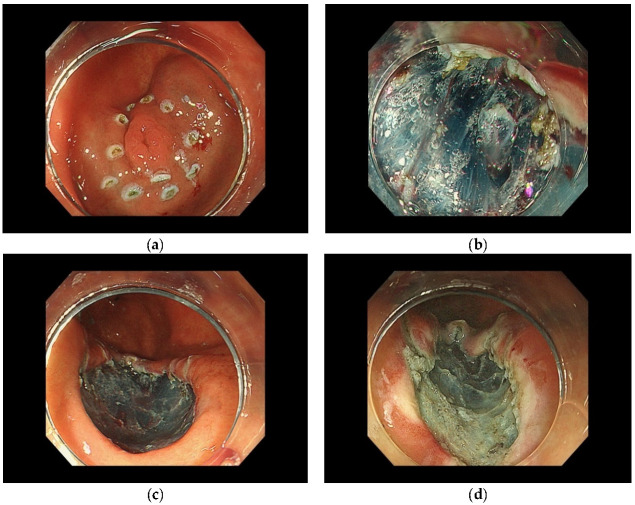
Method of ESD. (**a**) Markings located outside the horizontal margin of the lesion. (**b**) Submucosal tissue dissection beneath the lesion. (**c**) Endoscopic complete resection. (**d**) The defect after coagulation of visible vessels remaining in the resection area.

ESD comprises several steps, as previously reported [34,35,36]. First, marking the border of the lesion is performed, with markings located approximately 5 mm outside the horizontal margin of the lesion. Close inspection of the lesion is required at this time, and the use of a magnifying endoscope is recommended when possible. This is followed by injection of a lifting solution such as sodium hyaluronate solution or saline around the lesion. Using an endoscopic dissection device, a mucosal incision should be performed around the markings as landmarks. Additional injection of a sodium hyaluronate solution or similar solution into the submucosa under the lesion should be performed as necessary to thicken the target submucosa in order to prevent intraoperative perforation. Submucosal tissue dissection beneath the lesion is performed with care to avoid tissue damage to the muscle layer and perform en bloc resection of the lesion. Thereafter, prophylactic hemostasis should be performed for visible vessels remaining in the resection area after ESD. (Figure 1).

### 2.6. Curability of Endoscopic Resection for Gastric Cancer

According to current guidelines, endoscopic curability is based on local factors and risk factors for lymph node metastasis. Endoscopic curability (eCura) is divided into eCuraA, B, and C (Table 2).

#### 2.6.1. eCuraA

eCuraA is defined as treatment results with equal or superior long-term outcomes compared to additional surgical resection. When the lesion is resected en bloc with tumor-negative margins, the following conditions, (1) UL0 predominantly well-differentiated intramucosal cancers regardless of size without lymphatic or venous invasion (Ly0, V0); (2) UL0 predominantly undifferentiated intramucosal cancers less than 20 mm in diameter (Ly0, V0); or (3) UL1 well-differentiated intramucosal cancers less than 30 mm in diameter (Ly0, V0), are considered eCuraA [8].

#### 2.6.2. eCuraB

eCuraB is defined as treatment results for which sufficient evidence on long-term results has not been obtained, but curability can be expected. Specifically, predominantly differentiated adenocarcinoma with sm1 invasion, size ≤ 30 mm with Ly0, V0, HM0, and VM0 is considered eCuraB [8].

#### 2.6.3. eCuraC

eCuraC indicates treatment results that do not fit into either eCuraA or eCuraB and may require additional treatment. Differentiated type lesions that do not meet the criteria for eCuraA or eCuraB only due to positive lateral margin or non-en bloc resection are classified as eCuraC-1, while others are classified as eCuraC-2. In eCuraC-1, surgery, ESD, diathermy, or follow-up may be considered, depending on the institution’s policy. eCuraC-2 is subject to additional surgical resection [8]. However, there are many patients in which surgery cannot be performed due to the patient’s condition, and a multicenter retrospective study in Japan showed that 905 of 1969 eCuraC patients (46%) were selected for follow-up [39]. Hatta et al. proposed a risk assessment for lymph node metastasis. When the tumor size was >30 mm, VM1, V1, and submucosal invasion of more than 500 µm were defined as 1 point each, and Ly1 was defined as 3 points, lymph node metastasis was observed in 2.5% of patients in the low-risk group (total 1–3 points), 6.7% of patients in the intermedia group (2–4 points), and 22.7% of patients in the high-risk group (5–7 points) [40]. Based on the risk of lymph node metastasis and the patient’s general condition, the patient, surgeon, and gastroenterologist discuss and decide whether to perform additional surgery.

## 3. Complication of ESD

The main complications of ESD for EGC are postoperative hemorrhage and perforation. A large-scale Japanese multicenter prospective study for 10,821 cases of ESD for EGC reported complication rates of 4.4%, 0.7%, 2.3%, and 0.4% for postoperative hemorrhage, postoperative hemorrhage requiring blood transfusion, intraoperative perforation, and delayed perforation, respectively [41]. While most of these complications can be managed conservatively, preparation of endoscopic devices for the management of complications should they occur and cooperation with surgeons in the case of severe adverse events is necessary [42]. For the introduction of ESD to hospitals with no previous experience, training under the supervision of an expert is recommended to prevent complications [42]. Under expert supervision, it is suggested that there is no significant difference in complication rates between trainees and supervisors, while it is reasonable for a trainee to experience 30 cases before they can perform ESD with sufficient resection speed [43,44,45].

### 3.1. Postoperative Hemorrhage

Oda et al. reported that all cases of postoperative hemorrhage occurred within 14 days after ESD and that postoperative hemorrhage from lower gastric lesions tended to occur earlier than bleeding from the upper stomach [46]. In a meta-analysis, postoperative hemorrhage was reported to occur at a rate of 5.1% (95% CI, 4.5–5.7%). Risk factors of postoperative hemorrhage include male sex, cardiovascular disease, anticoagulants, cirrhosis, chronic kidney disease, tumor size > 20 mm, resection size > 30 mm, localization in the lesser curvature, flat and depressed type, carcinoma histology, and ulceration [47]. As for the prevention of postoperative hemorrhage, in a prospective randomized controlled trial, a proton pump inhibitor (PPI) was shown to be superior to an H2-receptor antagonist in preventing postoperative hemorrhage. Moreover, the usefulness of coagulation of visible vessels remaining in the resection area after ESD has been reported. This method has been established as post-ESD coagulation (PEC), and Takizawa et al. reported that the postoperative hemorrhage rate was reduced from 7.4% to 3.2% by PEC in their analysis of 1083 lesions [48].

Although second-look endoscopy has been widely used in the past, randomized controlled trials and meta-analyses have shown no significant difference in postoperative hemorrhage rates, and current guidelines for ESD and EMR indicate that second-look endoscopy after ESD is not necessary [8,49,50]. 

Methods such as mucosal closure with a detachable snare and clips, with an over-the-scope clip, and with endoscopic hand-suturing have been reported as having promise for postoperative hemorrhage prevention [51,52,53].

### 3.2. Perforation

Procedure time, lesions in the upper stomach, and tumor size are known to be risk factors for perforation [54,55]. In a retrospective study, 115 of 117 patients with perforation during EMR for EGC were successfully treated with endoscopic endoclips [56]. Therefore, when the perforation is identified during the endoscopic procedure, endoscopic closure is first attempted. Of course, even now, emergency surgery may be required after gastric perforation, and although rare, peritoneal dissemination after gastric perforation during ESD for EGC may occur, requiring careful follow-up [57].

## 4. Future Perspectives 

ESD is the first-line treatment for EGC in current guidelines [8,9,21]. However, there are some issues to be resolved, including topics such as screening for GC on a global level, implementation of ESD in Western countries with a low incidence of GC, endoscopic detection and diagnosis of EGC after *H. pylori* eradication, and optimizing indications of endoscopic treatment for the elderly. 

Especially, there is a large difference in the incidence of GC in various regions, and this is one of the main factors prolonging the establishment of a screening program for GC and implementation of ESD in countries with a low incidence of GC. While many countries with a high prevalence of *H. pylori* infection could greatly benefit from a screening program for GC, issues such as cost, medical insurance, and other social and economic problems must be overcome to implement a screening program on a national or global level. As for the implementation of ESD in countries with a low incidence of GC, while it has been reported that ESD could be safely and usefully performed in Western countries, the establishment of training methods is also an issue [58]. ESGE guidelines recommend performing at least 10 ESD procedures under expert supervision and 25 ESD procedures per year to maintain ESD performance [42]. However, these goals may not be easily reached in countries with a low incidence of GC [59]. These issues are complex and may not be resolved in the near future. 

Concerning endoscopic detection and diagnosis of EGC after *H. pylori* eradication, it is estimated that 10 million people in Japan received *H. pylori* eradication therapy between 2000 and 2016, and with the declining incidence of GC, the proportion of EGC after *H. pylori* eradication is increasing [60]. EGC after *H. pylori* eradication is generally characterized by smaller size, non-cardiac location, and microscopic depressed type [61,62]. However, the possibility that *H. pylori* eradication may impede early detection of GC has also been suggested, with a significantly higher prevalence of EGC with submucosal invasion after *H. pylori* eradication compared to the non-eradicated group [63]. For these reasons, regular endoscopy is important after *H. pylori* eradication, but establishing a useful method for detecting EGC after *H. pylori* eradication is an issue [64]. 

With the aging of the population, ESD is increasingly being performed for lesions which are diagnosed as a relative indication in elderly patients who are in poor condition and may not be able to tolerate surgical treatment. As mentioned above, endoscopic curability is assessed by risk factors for lymph node metastasis because lymph node metastasis of GC is extremely important regardless of the clinical stage [8,21,65]. However, the risk of acceptable lymph node metastasis may be different in patients in poor condition because of the high risk of complications from additional treatment and limited prognosis [66,67]. Therefore, the indication in the elderly is currently under investigation in multi-institution prospective trials (JCOG 1902). The main objective of study JCOG1902 is to determine whether follow-up is acceptable after ESD for EGC with a lymph node metastasis risk of 10% or less in patients aged 75 years or older in men and 80 years or older in women. This trial is expected to provide a basis for expanding the range of indications for early gastric cancer in the elderly.

## 5. Conclusions

This review described the present status and future perspective of endoscopic treatment of GC. It is important to understand the indications and curability for ER of EGC.

## Figures and Tables

**Table 1:** Indications of endoscopic resection of gastric cancer *^,^**.

Depth of Invasion	Ulceration	Differentiated Type		Undifferentiated Type	
cT1a(M)	UL0	**≤20 mm diameter absolute indications for EMR/ESD** ***	**>20 mm diameter** **absolute indications for ESD**	**≤20 mm diameter** **absolute indications for ESD**	>20 mm diameter Relative indications
	UL1	**≤30 mm diameter absolute indications for ESD**	>30 mm diameter Relative indications	Relative indications	
cT1b(SM)		Relative indications		Relative indications	

* Guidelines for endoscopic submucosal dissection and endoscopic mucosal resection for early gastric cancer (second edition). [8] Absolute indications are shown in bold. ** T1a/T1b, UL0/1 are as defined in Guidelines for endoscopic submucosal dissection and endoscopic mucosal resection for early gastric cancer (second edition) [8] *** EMR: endoscopic mucosal resection, ESD: endoscopic submucosal dissection.

**Table 2:** Curability of endoscopic resection of gastric cancer *^,^**.

Depth of Invasion	Ulceration	Differentiated Type		Undifferentiated Type	
cT1a(M)	UL0	**eCuraA** ***		**≤20 mm diameter** **eCuraA**	>20 mm diametere CuraB
	UL1	**≤30 mm diameter eCuraA**	>30 mm diameter eCuraC-2	eCuraC-2	
cT1b(SM1)		≤30 mm diameter eCuraB	>30 mm diameter eCuraC-2	eCuraC-2	
cT1b2(SM2)		eCuraC-2		eCuraC-2	

* Guidelines for endoscopic submucosal dissection and endoscopic mucosal resection for early gastric cancer (second edition). [8] eCuraA is shown in bold. ** T1a/T1b, UL0/1 are as defined in Guidelines for endoscopic submucosal dissection and endoscopic mucosal resection for early gastric cancer (second edition) [8] *** eCura: endoscopic curability.

## Data Availability

Not applicable.
